# How Do Women Interpret the NHS Information Leaflet about Cervical Cancer Screening?

**DOI:** 10.1177/0272989X19873647

**Published:** 2019-09-26

**Authors:** Yasmina Okan, Dafina Petrova, Samuel G. Smith, Vedran Lesic, Wändi Bruine de Bruin

**Affiliations:** Centre for Decision Research, Leeds University Business School, University of Leeds, Leeds, UK; Cancer Registry of Granada, Andalusian School of Public Health, Granada, Spain; Instituto de Investigación Biosanitaria de Granada (ibs.GRANADA), University of Granada, Spain; CIBER of Epidemiology and Public Health (CIBERESP), Madrid, Spain; Leeds Institute of Health Sciences, University of Leeds, UK; Centre for Decision Research, Leeds University Business School, University of Leeds, Leeds, UK; Centre for Decision Research, Leeds University Business School, University of Leeds, Leeds, UK; Department of Engineering and Public Policy, Carnegie Mellon University, Pittsburgh, PA, USA

**Keywords:** cancer screening, cervical cancer, informed decision making, patient information, risk communication

## Abstract

**Background.** Organized screening programs often rely on written materials to inform the public. In the United Kingdom, women invited for cervical cancer screening receive a leaflet from the National Health Service (NHS) to support screening decisions. However, information about screening may be too complex for people to understand, potentially hindering informed decision making. **Objectives.** We aimed to identify women’s difficulties in interpreting the leaflet used in England and negative and positive responses to the leaflet. **Methods.** We used a sequential mixed-methods design involving 2 steps: cognitive think-aloud interviews (*n* = 20), followed by an England-wide survey (*n* = 602). Data were collected between June 2017 and December 2018, and participants included women aged 25 to 64 y with varying sociodemographics. **Results.** Interview results revealed misunderstandings concerning screening results, benefits, and additional tests and treatment, although participants tended to react positively to numerical information. Participants were often unfamiliar with the potential harms associated with screening (i.e., screening risks), key aspects of human papillomavirus, and complex terms (e.g., *dyskaryosis*). Survey results indicated that interpretation difficulties were common (*M* correct items = 12.5 of 23). Lower understanding was associated with lower educational level (β’s >0.15, *P*’s <0.001), lower numeracy scores (β = 0.36, *P* < 0.001), and nonwhite ethnicity (β = 0.10, *P* = 0.007). The leaflet was evaluated positively overall. **Conclusions.** Despite previous user testing of the leaflet, key information may be too complex for some recipients. As a consequence, they may struggle to make informed decisions about screening participation based on the information provided. We discuss implications for the improvement of communications about screening and decision support.

Cervical cancer is highly preventable and typically caused by the human papillomavirus (HPV). HPV infection can cause abnormal changes in cervical cells, potentially leading to cancer. The primary aim of cervical screening is to detect abnormal cells, which can be removed before becoming cancerous. In the United Kingdom, population-based cervical screening was implemented in 1988 and has contributed to substantial reductions in cervical cancer incidence and cancer-specific mortality.^1–5^ In 2014, the age-standardized incidence was estimated at 11.8 in 100000, and the age-standardized mortality rate was 3.3 in 100 000.^6^ Recent estimates indicate that without screening, there would be 1827 additional cervical cancer deaths per year in England.^5^

The UK’s National Health Service (NHS) offers free cervical screening every 3 y to women aged 25 to 49 y and every 5 y to women aged 50 to 64 y. Eligible women are mailed an invitation letter and a leaflet containing information about cervical cancer, its causes, what screening involves, possible results, as well as screening benefits and risks. Benefits include reduction of cervical cancer incidence and mortality. Risks include potential detection and treatment of abnormal cells that would have cleared up on their own^7,8^ and increased risk of preterm birth among women who are treated to remove abnormal cells.^9–11^ Initial screening results are communicated by letter, and women invited for further tests (i.e., a colposcopy) receive an additional leaflet describing the procedure, possible results, and risks of treatment.

Besides raising awareness of cervical screening, a key aim of the invitation leaflet for England^i^ is to support informed choices about participation.^12^ However, communications about screening often involve quantitative information that can be complex, even for educated audiences.^13,14^ Concepts such as overdiagnosis and overtreatment are unfamiliar and counterintuitive to most people.^15,16^ Even NHS materials that have been user tested may include complex numerical information or terminology.^17^ Screening communications that are not well understood may cause undue concern, reduce recipients’ beliefs about their capability to participate in screening (i.e., self-efficacy), and undermine informed uptake.^13,18^ Individuals with lower levels of educational attainment or numeracy may be particularly affected, contributing to socioeconomic inequalities in screening participation.^19,20^

Here, we aimed to assess women’s difficulties in interpreting the NHS cervical screening leaflet for England. We also sought to explore women’s responses to the leaflet, including its numerical information and infographics. These aims were of relevance because the leaflet was being revised to reflect the move to HPV primary screening in England, whereby samples will first be tested for HPV.^21^ A better understanding of the weaknesses and strengths of the current leaflet can help to inform new versions and point to specific aspects requiring attention.

We used a sequential mixed-methods design involving 2 steps.^22,23^ First, qualitative cognitive think-aloud interviews aimed to identify women’s responses to and potential difficulties with the leaflet. Second, a quantitative survey aimed to examine the generalizability of interview findings by assessing the prevalence of difficulties and responses in the population. The survey also explored whether difficulties and responses varied with participant characteristics, including sociodemographics, screening experience, and numeracy. Participants in both steps were recruited from England, because the leaflet we tested focused on England. Ethical approval for both steps was obtained from the ethics committee of the University of Leeds (AREA 16-071 and AREA 17-002). All materials and survey data are available from the Open Science Framework (https://doi.org/10.17605/OSF.IO/8WQZV).^24^

## Step 1 Methods: Cognitive Think-Aloud Interviews

In cognitive think-aloud interviews, women were asked to vocalize their thoughts while reading the leaflet. This method provides access to the cognitive processes that occur during a task and is often used to identify potential usability problems.^17,25,26^

### Participants

Women were recruited in June 2017 via Luto Research Ltd. in Leeds, England. Our sample size (*n* = 20) was based on related think-aloud research^17^ and evidence that 10 to 15 interviews are typically enough to identify most usability issues or themes.^27,28^ Four pilot interviews were undertaken before the main 20. Luto telephoned potential participants from their database. Women were eligible if they were aged 25 to 64 y and had not had cervical cancer. Purposive sampling ensured diversity in age and education. Following Luto’s standard procedures, we excluded people taking medication for opioid addiction (due to potentially impaired cognitive function), current or retired health care professionals, and others routinely working with medical information.

### Leaflet

Participants received the leaflet titled “NHS Cervical Screening: Helping You Decide” (May 2017 version), which is available from https://www.webarchive.org.uk/wayback/en/archive/20170407074808/https:/www.gov.uk/government/publications/cervical-screening-description-in-brief.

### Procedure

Interviews were conducted in university meeting rooms by the first author. After giving informed consent, participants received standardized instructions about the think-aloud task. We used a marked protocol that instructed participants to read out the leaflet and think aloud every time they encountered a red asterisk in the text. Asterisks were placed at the end of bullet points, short paragraphs (i.e., 2 short sentences), and long sentences (i.e., more than 25 words).^17,25,ii^

**Figure 1:**
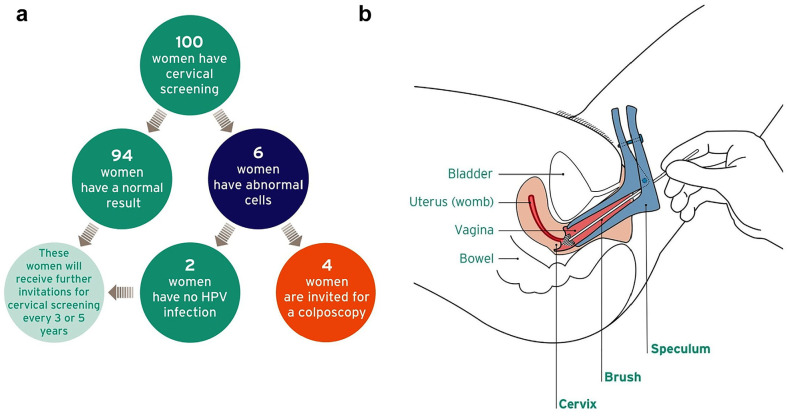
Images in the leaflet. (a) Infographic showing possible screening results. (b) Image depicting how the speculum is inserted. Reprinted with permission from Felton Works. Originally published in the leaflet “NHS Cervical Screening: Helping You Decide,” created by Public Health England on behalf of the National Health Service.

Following recommended procedures, participants first practiced with a leaflet about an unrelated topic.^26^ After 3 successful utterances, they received the cervical screening leaflet. Following the think-aloud task, participants answered questions about the leaflet, including how much they liked it, its numerical information, and the infographic of possible screening results (Figure 1a). Finally, they completed a questionnaire assessing participant characteristics, including cervical screening experience, previous abnormal results, knowledge of someone diagnosed with cervical cancer, first language (English or other), and ethnicity (Table 1). Participants also completed Schwartz et al.’s 3-item numeracy measure,^14^ which can provide good discriminability in samples of the general population. Details on age, education, and employment status were obtained from Luto.

**Table 1:** Participant Characteristics

	Interviews, Step 1 (*n* = 20)	Survey, Step 2^a^ (*n* = 602)
	*n* (%)	*n* (%)
Age (y)		
25–34	5 (25)	152 (25.2)
35–44	5 (25)	154 (25.6)
45–54	5 (25)	163 (27.1)
55–64	5 (25)	133 (22.1)
Mean (standard deviation), range (y)	44.8 (11.6), 26–62	44.4 (10.9), 25–64
Education		
≤GCSE/O-level grade or equivalent	8 (40)	294 (48.8)
A-levels or equivalent	8 (40)	90 (15.0)
Higher education or equivalent	4 (20)	218 (36.2)
Ethnicity		
White	20 (100)	532 (88.4)
Nonwhite	0 (0)	70 (11.6)
Social grade^b^		
AB (managerial/professional)	—	136 (22.6)
C1C2 (supervisory/skilled manual)	—	268 (44.5)
DE (semiskilled and unskilled manual/casualand lowest grade workers/unemployed)	—	198 (32.9)
First language		
English	20 (100)	543 (90.2)
Other	0 (0)	59 (9.8)
Numeracy score^c^		
0	7 (35)	92 (15.3)
1	2 (10)	180 (29.9)
2	5 (25)	213 (35.4)
3	6 (30)	117 (19.4)
Mean (standard deviation), range	1.5 (1.3), 0–3	1.6 (1.0), 0–3
Cervical screening experience		
Yes^d^	20 (100)	545 (90.5)
No	0 (0)	53 (8.8)
Previous abnormal result^e^		
Yes	7 (35)	145 (24.1)
No	13 (65)	389 (64.6)
Know someone diagnosed with cervical cancer^e^		
Yes	5 (25)	102 (16.9)
No	15 (75)	426 (70.8)

GCSE, General Certificate of Secondary Education.

a Sample sizes vary because of missing data. Percentages are calculated considering the total number of participants.

b Employment status but not social grade was recorded in step 1 (*n* = 14 employed, *n* = 4 unemployed or students, *n* = 2 retired).

c Numeracy was assessed using the measure by Schwartz et al.^14^ (skew step 1: –0.08; step 2: –0.12).

d Includes *n* = 5 who were overdue for screening in step 1 and *n* = 110 in step 2.

e In step 2, these questions were displayed only to participants who previously reported having screening experience.

### Analysis

Interviews were audio-recorded, transcribed verbatim, and analyzed in QSR NVivo12. We used thematic analysis—a qualitative approach for identifying relevant patterns of meaning, independently of quantifiable frequency measures.^29–31^ All transcripts were read by 2 researchers (Y.O. and D.P.). Y.O. generated initial codes and searched for initial themes and subthemes. Y.O. and D.P. reviewed themes and subthemes as needed and agreed on definitions and names. The thematic map was discussed iteratively with the remaining authors, who indicated whether the themes were adequately represented by the quotes and suggested alternative themes where relevant, until a final thematic map was defined.^17,iii^

## Step 1 Results

### Sample Characteristics

The sample (*n* = 20) was diverse in age, educational level, and numeracy, though all participants were of white ethnicity and had previously participated in cervical screening (Table 1).

### Themes

We identified 6 themes: 2 reflecting difficulties in interpretation, 2 reflecting negative reactions, and 2 reflecting positive reactions. Illustrative quotes are provided in Box 1.

**Box 1:** Themes Identified in Think-Aloud Interviews and Illustrative Quotes

Summary of Relevant Leaflet Sections	Example Quotes
**Misunderstandings and self-reported confusion: screening results, screening benefits, additional tests, and treatment**	
About half the women who have a colposcopy are found to have abnormal cells that need to be removed. (p. 8)	“So about half the women have abnormal cells. Six women have abnormal cells. 94 women have a normal result. Now don’t get me wrong, but that’s not half. That’s really not half. That’s really confused me.” (P19, 28 y, A-level qualifications)
Cervical screening helps prevent cervical cancer. It stops about 1 woman getting cervical cancer for every 100 women who have screening. (p. 9)	“Okay, so—okay. Because, of course, 96 were—No. 94 were okay and 6 had abnormal, and of that, only one person is likely to get cervical cancer out of the whole thing. Okay, that makes a lot of sense.” (P10, 45 y, GCSE-level qualifications)
Depending on the result of your test, your sample may be tested for the types of human papillomavirus (HPV) that can cause cervical cancer. (p. 3)	“So it’s letting me know that if you had any abnormal cells, they will be doing a sample of the test and testing it to see if you have got cervical cancer.” (P8, 42 y, GCSE-level qualifications)
**Knowledge gaps and unfamiliar concepts: HPV, screening risks (premature labor), colposcopy, dyskaryosis, and cervical screening (v. smear test)**	
HPV is found on the skin around the whole genital area, and can be spread through any type of sexual activity. This means that condoms do not always protect you from getting an HPV infection. (p. 11)	“It doesn’t tell me really who’s the carrier. Is it the man? Is it the woman? I’m not sure. And if it’s the man, does he have it for a while? Does it affect me in any way? If the woman gets cervical cancer, does the man get any kind of cancer from it? Testicular maybe? I don’t know.” (P3, 48 y, GCSE-level qualifications)
Women who get pregnant after having abnormal cells removed are slightly more likely to have their baby 1 to 2 months early. (p. 9)	“Maybe could do with some figures there because obviously that is a risk, and I know we have to be informed of risks, but I think that might just need putting into context a little bit, the numbers.” (P14, 48 y, A-level qualifications)
As a next step you may be offered another test (called a colposcopy) to look at your cervix more closely. If the person carrying out the colposcopy finds abnormal cells, they will suggest that you have the cells removed, usually during another colposcopy. (p. 3)	“Gosh, so what’s a colposcopy? What does that take? What does that involve, I would wonder. And the thought is, you may have to have it done twice. Quite a scary thought, maybe.” (P10, 45 y, GCSE-level qualifications)
A few women will have very abnormal cells in their sample. This is called high-grade dyskaryosis. If you have very abnormal cells, you will offered a colposcopy to check your cervix more closely. (p. 6)	“I’d like to know what that is, because it’s saying very abnormal cells and it’s a bit scary, and especially using big massive words, like the first thing you’re going to do is go on the internet and like look it up, so it’d be nice that it’s in there with that.” (P1, 38 y, A-level qualifications)
Cervical screening (which used to be called the ‘smear test’) involves taking a small sample of cells from the surface of your cervix. (p. 3)	“I think smear test is probably a better word because people might understand that. Why not keep the same name?” (P11, 60 y, higher educational qualifications)
**Concern about speculum insertion and pain**	
A device called a speculum will be put into your vagina and then used to open it gently. This allows the nurse or doctor to see your cervix. (p. 4)	“I don’t know whether all the speculums now are plastic but I think when I first had one it was a metal one and it was horrible. So I think it might be useful to put in there if it’s always going to be plastic speculums now.” (P7, 34 y, higher educational qualifications)
See Figure 1b (image depicting speculum). (p. 5)	“That does look painful to me, even though they’re saying it’s not, that looks horrible. It looks like a medieval torture device! [Laughs] No, I don’t like that.” (P6, 40 y, higher educational qualifications)
**Disagreement with screening eligibility and frequency**	
The NHS offers cervical screening to all women aged 25 to 49 every 3 years and to all women aged 50 to 64 every 5 years. This is because most cervical cancers develop in women aged 25 to 64. (p. 1)	“I know to me it just screams like, well, it’s just cost cutting really because it’s just saying most happen in those age groups. But I think there could be a potential to save more if it wasn’t just restricted to those ages.” (P13, 51 y, A-level qualifications)
If you do not have an HPV infection, you have a low risk of developing cervical cancer before your next screening test. So you will be invited back for screening again in 3 or 5 years depending on your age, as usual. (p. 6)	“I still think it’s too long. It should be less than 3 years and 5 years. I think every year and a half really. I mean I’m not being funny but an eye test you’re called every 2 years. This is worse than an eye—things like that. It should be every year or every year and a half. I don’t know.” (P20, 41 y, GCSE-level qualifications)
**Positive reactions to statistical information about screening results and benefits**	
Out of 100 women who have cervical screening, about 94 will have a normal result. If you have a normal result, you will have a very low risk of developing cervical cancer before your next screening test. (p. 6)	“So that’s good because it is saying that most women, it’s innocent and it’s a good result, so that’s reassuring, you know, the numbers and your chances are it should be okay, so that’s good.” (P5, 62 y, GCSE-level qualifications)
Cervical screening saves as many as 5,000 lives from cervical cancer a year in the UK. (p. 9)	“So it’s a good point really. It’s basically promoting having a screening test, basically, because of how many lives it’s saved each year. It’d make me want to definitely go for a cervical screening test, knowing the facts like that.” (P12, 26 y, GCSE-level qualifications)
**Liking of information about the procedure**	
Cervical screening is usually carried out by a female nurse or doctor. If you want to make sure a woman carries out your test, you can ask for this when you make your appointment. (p. 4)	“Yeah, that’s good to know. People need to know that, because the worst thing that you—you don’t want to walk into a room and there’s a man nurse there or, do you know what I mean.” (P1, 38 y, A-level qualifications)
The nurse or doctor will ask you to undress from your waist down and lie on a bed with your knees bent and apart. (p. 4)	“I think that’s good information because it’s fully explaining everything so you can prepare yourself, knowing what’s going to happen when you get there so you’re not given the unexpected and are a bit scared about what’s going to happen.” (P12, 26 y, GCSE-level qualifications)
The actual test takes only a minute or two. The whole appointment usually takes about 10 minutes. (p. 4)	“I think that’s really good to mention, because it is only very quick and I think if you’re worried about it I think you need to know that it’s not necessarily going to take a long time at all, so that’s really good to mention.” (P16, 34 y, higher educational qualifications)

GCSE, General Certificate of Secondary Education; HPV, human papillomavirus; NHS, National Health Service.

#### Misunderstandings and self-reported confusion

This theme reflects aspects of the leaflet that were either not interpreted as intended or resulted in confusion. It included 3 subthemes.

##### Screening results

Numerical information about possible screening results caused confusion. For instance, the leaflet states that of 100 women who have cervical screening, about 94 will have a normal result, 6 will have abnormal cells, and 4 will be invited for a colposcopy. The leaflet further states that “about half the women who have colposcopy are found to have abnormal cells that need to be removed.” Thus, the leaflet implies that about 2 in 100 women will need treatment for abnormal cells. Instead, participants appeared to infer that half of the women who have screening may have abnormal cells.

##### Screening benefits

Participants also misunderstood numerical information about screening benefits. Specifically, the leaflet explains the reduction in the risk of getting cervical cancer by stating that “screening stops about 1 woman getting cervical cancer for every 100 women who have screening.” Some participants incorrectly inferred that this implies that 1 out of 100 women who have screening will be diagnosed with cancer.

##### Additional tests and treatment

Participants expressed confusion about the purpose of additional tests and when these may be offered. The leaflet explains that if slightly abnormal cells are detected, the sample will be tested for the HPV types that can cause cervical cancer. Some participants incorrectly inferred that samples would be tested for cancer if abnormal cells are detected. Others incorrectly inferred that treatment for abnormal cells is offered to women who test positive for HPV or abnormal cells, independently of colposcopy results.

#### Knowledge gaps and unfamiliar concepts

This theme focuses on concepts that were unfamiliar to participants and in some cases were seen as concerning or scary. Participants often noted that additional clarifications about these concepts would be useful. This theme included 4 subthemes.

##### HPV

Participants often noted that they were not previously aware of HPV, its link to cervical cancer, how it is transmitted, or the fact that it can regress without treatment. Some participants wondered how HPV might affect men.

##### Screening risks

Some participants expressed concern about the risk of premature labor associated with treatment for abnormal cells and noted that it would be good to quantify the risk.

##### Complex terms (colposcopy, dyskaryosis)

Participants often struggled to pronounce these terms and highlighted their complexity. Some questioned what a colposcopy would involve and whether it would hurt, particularly after reading initial sections of the leaflet about this.

##### Cervical screening (versus smear test)

Some participants noted that they were more familiar with the term *smear test* to describe cervical screening.

#### Concern about speculum and pain

Participants noted that the procedure might be uncomfortable or painful. Some mentioned their own unpleasant experiences with the speculum. Several also found the image of how the speculum is inserted off-putting (Figure 1b).

#### Disagreement with screening eligibility and frequency

Participants generally questioned the current age range for screening and felt that screening should start earlier or end later. Some also noted that screening should be more frequent. Participants’ views on these issues were generally strong, despite a seemingly limited awareness of the rationale behind the current recommendations.

#### Positive reactions to statistical information about screening results and screening benefits

Despite some misunderstandings, participants tended to react positively to statistical information about screening benefits. For instance, the leaflet also mentions that cervical screening saves 5000 lives from cervical cancer each year in the United Kingdom. This information was often viewed as encouraging. Participants noted that it highlighted the importance of screening. In addition, information about screening results was often viewed as reassuring.

#### Liking of information about the procedure

Participants noted that the information about the procedure and specific advice on how to prepare for the test were useful. They emphasized that it was good to be informed of the expected length of the appointment, waiting time to receive initial results, and of the option to ask that a woman performs the test.

### Leaflet Evaluations

The leaflet was evaluated positively, with a mean rating of 5.9 (SD = 1.0) on a scale ranging from 1 to 7. Evaluations were also often positive for the numeric information (M = 5.8, SD = 1.5) and the infographic showing screening results (M = 6.1, SD = 1.3)

## Step 2 Methods: Survey

### Participants

Survey respondents were recruited through research company Norstat in December 2018. Norstat e-mailed invitations to potentially eligible individuals in their database who could speak English to a native standard. Women were eligible if they were aged 25 to 64 y, lived in England, and had not had cervical cancer or a hysterectomy.^32,33^ We excluded those who reported being registered with a general practitioner in a location where HPV primary screening was piloted at the time, because that experience could potentially interfere with interpretations of the leaflet (see Supplementary Table S1 for a list of pilot sites). We set quotas for age, education, and ethnicity, taking into account distributions in the target population of English women aged 25 to 64 y (Supplementary Table S2). The survey was first piloted with 20 participants. The target sample size for the main survey (*n* = 601) was set to estimate the prevalence in the target population (*n* = 14133497),^34^ with a confidence level of 95% and a margin of error of 4%. Following standard practice, Norstat overrecruited to meet the target sample size after removing inattentive participants who completed the survey in less than half of the median completion time (median time = 18 min 11 s).

### Leaflet

The leaflet was the same as in step 1, with the exception that we removed 3 sections that were not linked to interpretation difficulties in step 1 to avoid excessive respondent burden: 1) the procedure and specific advice on how to prepare, 2) the symptoms of cervical cancer, and 3) storage of samples after screening.

### Survey Items

Items assessing interpretations were built on the first 2 themes identified in step 1 (Box 1). We also developed items for each of the remaining themes, except for the sixth theme (i.e., liking of information about the procedure), as the corresponding information was removed from the leaflet (see above).

#### Interpretations

We developed items for each subtheme under “misunderstanding and self-reported confusion” and “knowledge gaps and unfamiliar concepts”. We also developed items assessing understanding of other aspects relevant for screening decisions, including additional screening risks (overtreatment, false positives, false negatives) and the main goal of cervical screening.^35–37^ Items were pretested iteratively using 3 rounds of cognitive interviews conducted by the first author (*n* = 4 per round, 12 in total). Participants thought aloud while answering each item and were probed for further details where relevant. They also suggested alternative wording for items that were unclear or confusing.^38^ Following each pilot round, items were revised with all authors to reduce reading barriers and ensure that they were interpreted as intended. The final set of items included 19 true/false items (10 true and 9 false) and 4 open-ended items.^iv^ All items are shown in Table 2. For each item, participants expressed their confidence in their answers on a scale ranging from 50% (just guessing) to 100% (absolutely sure).^42^

**Table 2:** Survey Results for Items Assessing Interpretations^a^

Item	% Correct	Mean Confidence
Screening results		
Imagine 1000 women who have cervical screening. About how many of them will . . .		
Have an abnormal result? (60)	43.2	90.1
Need treatment to remove abnormal cells? (20)	15.3	73.9
Have cells that could be cancer? (1)	10.3	82.7
Additional tests and treatment		
If a woman has slightly abnormal cells, her sample gets tested for cancer next. (F)	46.2	87.6
If a woman has HPV, she is offered treatment to prevent cancer. (F)	41.4	83.8
If a woman has very abnormal cells, she is offered treatment to prevent cancer. (F)	30.4	86.7
Screening benefits and main goal of cervical screening		
Cervical screening prevents as many as 5000 cervical cancer deaths each year in the United Kingdom. (T)	96.7	91.3
Cervical screening lowers the risk of getting cervical cancer. (T)	94.4	93.9
The main goal of cervical screening is to find cancer that is already there. (F)	73.6	89.9
Among 1000 women who do not have cervical screening, about 20 will get cervical cancer. Now imagine 1000 women who do have cervical screening. How many do you think will get cervical cancer? (10)	34.7	71.9
In 1 out 100 women, cervical screening helps to find cancer that is already there. (F)	32.7	86.8
Screening risks		
A woman who does not have abnormal cells could get an abnormal test result. (T)	69.8	80.4
Cervical screening can lead to treatment of abnormal cells that is not needed. (T)	53.2	82.6
Imagine a woman has a cervical screening test. If she gets pregnant later, it is slightly more likely that her baby will be born early. (F)	25.1	91.7
A normal test result rules out that there are any abnormal cells (F)	24.4	84.8
HPV		
HPV can be passed on during sexual intercourse. (T)^b^	91.0	92.6
Men can’t get HPV. (F)^b^	72.4	82.1
HPV is a sexually transmitted infection (STI). (T)	66.4	88.6
HPV usually doesn’t need any treatment. (T)^b^	56.8	85.8
Using condoms lowers the risk of getting HPV. (T)^b^	49.8	89.3
Colposcopy, dyskaryosis		
A colposcopy checks if there are abnormal cells in the cervix. (T)	89.7	91.8
Cervical cancer cells are known as “high-grade dyskaryosis.” (F)	33.9	81.2
Cervical screening (v. smear test)		
Cervical screening tests were previously known as smear tests. (T)	99.0	97.5

F, false; HPV, human papillomavirus; T, true.

a Correct answers (based on the information in the leaflet) are indicated in brackets.

b Items adapted from the measure of knowledge about HPV by Waller et al.^46^

#### Evaluations of image depicting speculum

We assessed evaluations of this image (Figure 1b) in relation to the theme “concern speculum inserted and pain.” We adapted 3 items from previous work^43^ (e.g., “How much do you like or dislike this image?”) using a response scale ranging from 1 to 7 (e.g., 1 = *do not like it at all*, 7 = *like it a lot*). We averaged across items to produce an overall evaluation score (Cronbach’s α = 0.70). We also asked participants to indicate how the image affected their motivation to attend screening when next invited using 3 response options: “it decreases/increases/does not affect my motivation”

#### Views on screening eligibility and frequency

We developed 3 items assessing views on the current starting age, ending age, and frequency (e.g., “I think screening should start . . . at 25/before 25/after 25”). Participants who expressed disagreement with current policy (e.g., who selected “before 25”) were also asked to specify their preference (e.g., “At what age do you think screening should start?”).

#### Evaluations of infographic showing screening results

We assessed evaluations of this infographic (Figure 1a) in relation to the theme “positive reactions to statistical information about screening results and screening benefits.” Items were analogous to those assessing evaluations of the image depicting the speculum, described above (Cronbach’s α = 0.88). Participants also indicated how the infographic affected their motivation to attend screening.

#### Overall leaflet evaluations and familiarity

We developed 3 items to assess overall evaluations of the leaflet (Cronbach’s α = 0.83). In addition, we included an item to assess participants’ familiarity with the leaflet (i.e., whether they had read it before).^44^ Results for all individual evaluation items in the survey are presented in the supplement (Supplementary Table S3).

### Procedure

The survey was implemented in Qualtrics. Participants first read an online consent form. Those who agreed to proceed were then presented with questions assessing eligibility. In addition to the sociodemographics recorded in step 1, step 2 also assessed participants’ social grade according to the National Readership Survey system.^45^ Categories represented the occupation of the chief income earner of the household (Table 1). Next, participants viewed the leaflet and answered items assessing interpretations. The different pages of the leaflet appeared on separate screens, accompanied by the corresponding interpretation items immediately below. Next, they completed items assessing leaflet evaluations, familiarity with the leaflet, and views on screening eligibility and frequency. They were then presented with the image depicting the speculum (Figure 1b), the infographic showing screening results (Figure 1a), and associated items in each case. Finally, they completed questions assessing participant characteristics analogous to those in step 1, including the same numeracy measure.^v^

### Analysis

We computed overall accuracy scores for each participant by adding the number of correct responses to all items assessing interpretations. Missing responses were coded as incorrect. We performed multiple (univariate) linear regression analyses to examine whether accuracy scores, mean confidence ratings, and leaflet evaluations varied as a function of participant characteristics. Predictors consisted of sociodemographics (age, education, ethnicity, and social grade), cervical screening experience, numeracy, and English as a first language.^vi^ The lowest educational level and social grade were used as the reference class, and age and numeracy scores were entered as continuous variables. Analyses were conducted using SPSS 23 for Windows. Full regression results are presented below, and mean accuracy, confidence, and evaluations corresponding to the different levels of all predictors are presented in the supplement (Supplementary Table S4).

## Step 2 Results

### Sample Characteristics

**Figure 2:**
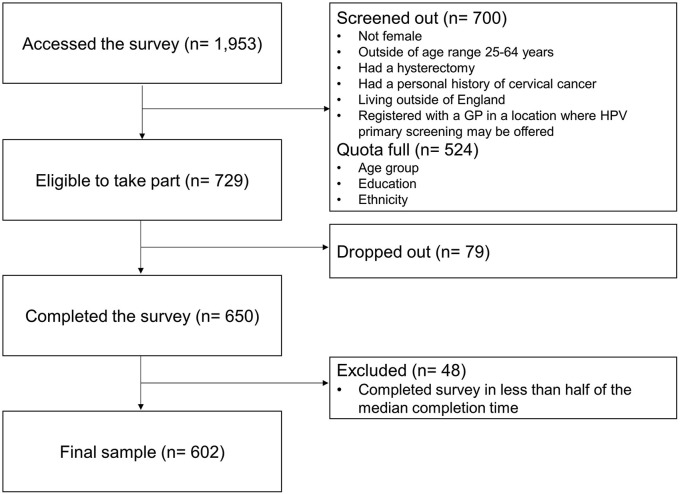
Overview of recruitment in step 2 (survey).

The survey was accessed by 1953 participants, of which 37% were eligible (Figure 2). The final sample (*n* = 602) included 12% participants of nonwhite ethnicity and 9% with no screening experience (Table 1). In the population, 14% were nonwhite and 11% has no screening experience (Supplementary Table S2).

### Interpretations

Participants answered on average 12.5 items correctly out of 23 (SD = 3.06; range, 5–21), indicating relatively common interpretation difficulties. Regression results revealed that the strongest predictor of accuracy was numeracy, followed by education (Table 3). Scores were lower among participants with GCSE/O-level grade or less, relative to participants with A-levels and to those with higher education. Accuracy was also lower among nonwhites than among whites and among participants from the lowest social grades (D and E) relative to those from grades C1 and C2.

**Table 3:** Linear Regression Models Predicting Accuracy of Interpretations, Self-Reported Confidence, and Overall Leaflet Evaluations

	Accuracy of Interpretations (0–23)			Self-Reported Confidence (50–100)			Leaflet Evaluations (1–7)		
	*B* (*SE*)	β	*P*	*B* (*SE*)	β	*P*	*B* (*SE*)	β	*P*
Age	0.01 (0.01)	0.04	0.317	0.10 (0.04)	0.11	0.006	−0.00 (0.00)	−0.03	0.462
Education									
GCSE/O-level grade or less v. A-levels	1.25 (0.35)	0.15	0.000	2.33 (1.23)	0.08	0.059	−0.05 (0.14)	−0.02	0.705
GCSE/O-level grade or less v. higher education	1.27 (0.29)	0.20	0.000	2.90 (1.01)	0.14	0.004	−0.03 (0.11)	−0.01	0.788
Ethnicity (1 = white; 0 = nonwhite)	0.98 (0.36)	0.10	0.007	0.85 (1.29)	0.03	0.509	0.02 (0.14)	0.01	0.878
Social grade									
DE (semiskilled/unskilled/unemployed)v. AB (managerial/professional)	0.44 (0.34)	0.06	0.197	1.96 (1.22)	0.08	0.107	0.22 (0.13)	0.09	0.109
DE (semiskilled/unskilled/unemployed)v. C1C2 (supervisory/skilled manual)	0.54 (0.27)	0.09	0.044	2.51 (0.95)	0.13	0.009	0.14 (0.11)	0.07	0.173
First language (1 = English; 0 = other)	−0.34 (0.40)	−0.03	0.387	0.87 (1.40)	0.03	0.536	0.33 (0.16)	0.09	0.036
Numeracy	1.13 (0.12)	0.36	0.000	1.39 (0.32)	0.14	0.001	0.00 (0.05)	0.00	0.998
Cervical screeningexperience (1 = yes; 0 = no)	−0.42 (0.40)	−0.04	0.288	3.46 (1.42)	0.10	0.015	0.52 (0.16)	0.14	0.001
Model statistics	*R*^2^ = 0.25*F*(9, 588) = 21.66, *P* < 0.001			*R*^2^ = 0.10*F*(9, 588) = 7.38, *P* < 0.001			*R*^2^ = 0.03*F*(9, 588) = 2.19, *P* = 0.021		

GCSE, General Certificate of Secondary Education.

Analyses of individual items revealed that performance was particularly poor for items assessing screening results (Table 2). Only 10% of participants accurately estimated the number of women expected to have possible cancer cells, and only 15% accurately estimated the number expected to need treatment for abnormal cells. Inspection of the distribution of responses revealed that participants often overestimated the likelihood of these adverse results (Supplementary Table S5a–c; Figure S1). For instance, 32% of participants inferred that 40 in 1000 women would have possible cancer cells (correct answer = 1 in 1000), and 19% inferred that 500 in 1000 women who have screening would need treatment for abnormal cells (correct answer = 20 in 1000). A different pattern emerged for the item concerning the number of women expected to have an abnormal result, where 43% of estimates were accurate and 48% were lower than the correct answer (60 in 1000). The fact that the correct answer for this item is higher than that of the previous 2 items implies that there was more room for underestimation. In addition, some incorrect responses likely reflect a failure to transform the estimate provided in the leaflet as required by the question. Whereas the leaflet stated that 6 out of 100 women will have an abnormal result, participants had to indicate how many out of 1000 would have an abnormal result. The most common incorrect response (seen in 31% of participants) was 6, which likely reflects direct extraction of the information from the leaflet.

Performance was also poor for items assessing understanding of additional tests and treatment. The most common misunderstandings were that treatment would be offered to women with abnormal cells (70% of participants) or those who test positive for HPV (57%). Instead, the leaflet explains that a colposcopy is offered in both cases to determine whether treatment is needed.

Information about screening benefits and risks was also misunderstood frequently, although performance varied substantially across individual items. Whereas most participants (94%) understood the concept that screening lowers the risk of getting cervical cancer, two-thirds (67%) misinterpreted the risk reduction information provided (“screening stops about 1 woman getting cervical cancer for every 100 women who have screening”). Only 35% of participants accurately estimated the effect of screening on the risk of getting cervical cancer, with 18% assuming that the risk would be equal in groups of unscreened versus screened individuals (Supplementary Table S5d). Moreover, 26% of participants assumed that the main goal of cervical screening was diagnosis rather than prevention. Concerning screening risks, one of the most common misunderstandings was that the screening test itself increased risk of premature labor (75% of participants). In addition, 76% were unaware of the possibility of false-negative results, and 47% did not understand that screening can lead to unnecessary treatment.

Specific aspects of HPV were also misinterpreted. Although most participants (91%) understood that HPV can be passed on during sexual intercourse, 50% incorrectly inferred that condoms do not lower the risk of infection. More than half (57%) also failed to understand that HPV usually does not need any treatment.

### Self-Reported Confidence

Despite participants’ misunderstandings, their self-reported confidence was relatively high (Table 2). Mean confidence ranged between 73.9 and 90.1 for items assessing screening results and additional tests and treatment, despite poor performance. Mean confidence across all items was weakly correlated with the total number of accurate responses (*r* = 0.21, *P* < 0.001), suggesting that participants who had better understanding tended to express more confidence. Regression results revealed that confidence ratings were higher among more numerate participants and among those with higher education, relative to those with GCSE/O levels or less. Confidence was also higher among participants from social grades C1 and C2, relative to those from the lowest grades (D and E). Older age and cervical screening experience were also associated with higher confidence.

### Image Depicting Speculum

This image (Figure 1b) was on average evaluated positively, with a mean rating of 5.2 (SD = 1.3) on a 1 to 7 scale. Most participants (72%) noted that their motivation to attend screening would not be affected by this image, although 14% noted that it would decrease their motivation, with the remaining 14% saying that it would increase it.

### Screening Eligibility and Frequency

Agreement with the current screening starting age (i.e., 25 y) and ending age (i.e., 64 y) was low (24% and 33% of participants, respectively). Most participants (72%) indicated that screening should start before age 25, of which 43% noted that it should start at 18 y. Most (64%) also indicated that screening should end after age 64, of which 47% stated that it should end at age 70. In addition, 35% participants indicated that screening should be offered more frequently, although 62% agreed with the current screening interval.

### Infographic Showing Screening Results

The infographic (Figure 1a) received very positive evaluations, with an average rating of 6.0 (SD = 1.1) on a 1 to 7 scale. A total of 31% participants noted that the infographic would increase their motivation to attend screening, with only 2% saying that it would decrease it.

### Overall Leaflet Evaluations and Familiarity

The leaflet overall was also evaluated positively, with a mean rating of 5.8 (SD = 1.1) out of 7. The regression predicting evaluations explained a small amount of variance (Table 2). Participants who had cervical screening experience and whose first language was English evaluated the leaflet more positively. Most participants (64%) reported having read at least some of the leaflet the last time they were invited for screening, although 18% reported not having read it, and the remaining 18% did not remember previously seeing a leaflet.

## Discussion

Our findings suggest that the NHS leaflet about cervical screening may be too complex for some recipients. Even though the leaflet underwent extensive user testing ^12^ and was evaluated positively in our study, we documented common misunderstandings about key aspects, including screening benefits, risks, and results. Despite these misunderstandings, participants’ self-reported confidence in their answers was relatively high. This echoes previous findings on overconfidence in one’s own knowledge^42,47,48^ (but see Olsson^49^). We also found that leaflet interpretations were less accurate among participants with lower education, lower numeracy, and ethnic minorities. These findings suggest that some recipients may struggle to make informed decisions about screening participation based on the information provided and highlight the challenges in developing communications that are effective for diverse audiences.

In addition to hindering informed decision making, specific misunderstandings may have other unintended effects. Although information about screening results was often viewed as reassuring by interviewees, survey respondents overestimated the likelihood of some adverse results. Relatedly, about a quarter of survey respondents failed to understand the preventive purpose of cervical screening, converging with recent findings.^50^ Misunderstanding of the main goal of cervical screening coupled with overestimations of adverse results may lead to undue worry about what the test might find. This in turn may potentially lead to avoidance of screening, particularly among women with high cancer fear.^50,51^ The misunderstanding that the screening test increases the risk of preterm labor could have a similar effect, particularly among those planning to get pregnant. On the other hand, we also found that almost half of the respondents failed to infer that cervical screening can lead to unnecessary treatment. The failure to understand the risk of overtreatment may lead to a more positive attitude about screening, at the expense of informed decision making.

Despite participants’ misunderstandings, they evaluated statistical information relatively positively. Indeed, there is evidence that numbers are often trusted and preferred over verbal quantifiers alone to communicate health risks.^52–54^ The finding that the infographic showing screening results (Figure 1a) was evaluated positively also converges with research showing that simple visual aids are often liked by diverse audiences.^55,56^ However, our findings also suggest that it may be beneficial to consider alternative numerical formats to support understanding. For instance, the leaflet did not provide information about the risks of developing cervical cancer and dying of cervical cancer with and without screening, contrasting with recommendations from the risk communication literature and International Patient Decision Aids Standards.^57^ Such information could be communicated in an accessible way using fact boxes and/or visual aids,^58–60^ which could facilitate evaluations of the effectiveness of screening. It could also be beneficial to add numerical estimates about screening risks, which are currently lacking in the leaflet. The use of verbal quantifiers without numbers to express risks is generally discouraged, as this can lead to diverse interpretations, including overestimations of risk.^61–63^

Our findings also show that unfamiliar concepts may not be fully understood based on the information in the leaflet. Misunderstandings about HPV are of particular concern considering the move to HPV primary screening. Our findings support work that has identified similar gaps in HPV knowledge,^64,65^ and provide the first evidence that some misunderstandings may persist despite the explanations provided in the leaflet. Hence, our findings highlight the importance of further clarifying key aspects of HPV, such as its link with cervical cancer, transmission, and how it can clear without treatment. In addition, the leaflet could potentially be simplified by removing other unfamiliar concepts that are arguably not essential for informed screening decisions at the invitation stage, such as “dyskaryosis” or specific aspects concerning colposcopies. Simplifying communication materials can increase understanding among diverse audiences without negatively affecting evaluations or intentions to participate in the advertised programs.^66^

### Limitations and Future Research

Our work has limitations. First, the marked think-aloud procedure may have introduced some bias as it encouraged comments at specific points in the text. Although prompts to think aloud were very frequent, they may have focused participants more on aspects immediately preceding each prompt. Second, our survey sample was recruited from an online panel, which may not have been representative of the population. Although we used quotas considering distributions of key demographics in the population, we were unable to recruit enough participants with no qualifications (8% in our sample v. 16% in the population; Supplementary Table S2). Relatedly, all think-aloud interviewees had previously participated in cervical screening at some point, which may have facilitated interpretations of information they could relate to their own experience. Some of this information (e.g., details about the procedure) was not tested in the survey because interviewees showed no confusion. Previous screening experience could potentially also result in more positive reactions to such information.

Third, although we took measures to remove inattentive survey participants, others may not have read the leaflet carefully either. However, any misunderstandings attributable to inattention may be present among actual leaflet recipients, who often do not read the full leaflet.^47^ In addition, it is also possible that performance was negatively affected by specific item wordings. Although we pretested all items, some may have not been interpreted as intended. For instance, the item concerning the risk of preterm labor may have been interpreted by some as referring to screening participation generally, rather than to the screening test itself. Relatedly, the item assessing estimates of the cervical cancer risk reduction associated with screening did not provide a time interval (e.g., lifetime risk). While a time interval was also lacking in the leaflet, this may have contributed to interpretation difficulties. The chances of correct responses due to guessing should also be considered when interpreting our results. The high confidence ratings suggest that participants did not report guessing in most cases. However, some may have been reluctant to admit doing so.

Future work could examine the impact of cultural differences and prior beliefs about cancer or screening on interpretations of cervical screening communications. Previous beliefs about the effectiveness of screening in general or strong fears from cancer could interfere with comprehension or its relationship to screening intentions.^59,67^ Similarly, low perceived cancer risk or cancer fatalism (e.g., the belief that cancer is incurable) could bias processing of information about screening, leading to misinterpretations. Such beliefs are more prevalent among ethnic minorities than among white British women, independently of other sociodemographic factors.^68,69^ This could help to explain our finding that nonwhite ethnicity was linked with lower leaflet understanding after controlling for other sociodemographics and native language.

## Conclusions

Our work points to strengths and weaknesses in the NHS cervical screening leaflet for England, which constitutes a central communication tool of the screening program. Addressing the weaknesses may contribute to reduce screening inequalities and support understanding for wider audiences. While we focused on the leaflet for England, our findings are also relevant for the design of other leaflets that may be revised to reflect the move to HPV primary screening (e.g., the Scottish leaflet). Our findings also have implications for improving other communications about cervical screening (e.g., Web sites), as well as potentially about other screening programs internationally.

## Supplemental Material

Cervical_screening_leaflet_R1_online_supp – Supplemental material for How Do Women Interpret the NHS Information Leaflet about Cervical Cancer Screening? Supplemental material, Cervical_screening_leaflet_R1_online_supp for How Do Women Interpret the NHS Information Leaflet about Cervical Cancer Screening? by Yasmina Okan, Dafina Petrova, Samuel G. Smith, Vedran Lesic and Wändi Bruine de Bruin in Medical Decision Making

## Footnotes

i. Each of the 4 countries of the United Kingdom—England, Scotland, Wales, and Northern Ireland—has its own leaflet. Specific content varies across leaflets. Here we focused on the leaflet for England.

ii. Marked protocols encourage reports of misunderstandings and confusion that may otherwise go unnoticed^25^ and have previously been used to examine comprehension of patient information leaflets.^17^ This procedure also proved more effective in our pilot interviews than unmarked protocols.

iii. This process reflects an established thematic analysis procedure involving 6 iterative phases^31^: 1) familiarization with the data, including repeated reading and noting initial ideas, 2) generating initial codes by systematically identifying patterns, 3) searching for themes by combining the initial codes into potential themes and subthemes (i.e., specific topics within a theme), 4) reviewing themes by checking that the coded quotes form a coherent pattern and that the thematic map reflects the meanings in the whole data set, 5) defining and naming themes, and 6) final analysis and write-up.

iv. We used the true/false format for 3 reasons. First, true/false items do not require considering multiple alternatives at a time and hence are less cognitively demanding than multiple-choice items.^39^ Second, true/false items are less likely to artificially increase the scores of test-wise respondents, who may use cues in the set of answer options in multiple-choice items (e.g., excess specificity of some options, length, or order of options).^40^ Third, true/false items can help to detect instances of mixed or partial understanding of a given concept.^41^

v. The survey also included an alternative image depicting the speculum and items unrelated to the current research questions (i.e., perceptions of the risk of developing cervical cancer and screening intentions), which will be reported elsewhere.

vi. We did not include previous abnormal results as a predictor because this question was answered only by participants who reported having screening experience. Because of an error in survey flow, this was also the case for the item assessing knowledge of someone diagnosed with cervical cancer, which was also not included as a predictor.
