# Supplementary material for: How Do Women Interpret the NHS Information Leaflet about Cervical Cancer Screening?
Source: Med Decis Making. 2019 Sep 26;39(7):738–54. doi: 10.1177/0272989X19873647 (PMC6843617; doi:10.1177/0272989X19873647)
Supplement: Cervical_screening_leaflet_R1_online_supp – Supplemental material for How Do Women Interpret the NHS Information Leaflet about Cervical Cancer Screening? [file Cervical_screening_leaflet_R1_online_supp.pdf]

Supplementary materials for Medical Decision Making

How do women interpret the NHS information leaflet about cervical cancer screening?

Yasmina Okan [1] Dafina Petrova [2,3,4] Samuel G. Smith [5] Vedran Lesic [1] Wändi Bruine de Bruin[1,6]

[1] Centre for Decision Research, Leeds University Business School, University of Leeds

[2] Cancer Registry of Granada, Andalusian School of Public Health

[3] Instituto de Investigación Biosanitaria de Granada (ibs.GRANADA), University of Granada

[4] CIBER of Epidemiology and Public Health (CIBERESP)

[5] Leeds Institute of Health Sciences, University of Leeds

[6] Department of Engineering and Public Policy, Carnegie Mellon University

Corresponding author: Yasmina Okan. Centre for Decision Research, Leeds University Business School, University of Leeds, Charles Thackrah Building, Leeds, LS2 9LB, UK; E-mail: y.okan@leeds.ac.uk

List of sites offering HPV primary screening

Table S1 shows the list of locations offering HPV primary screening at the time the survey (step 2) was conducted. Individuals who reported being registered in any of the locations listed were not invited to complete the survey, to ensure that participants had no experience with HPV primary screening.

Table S1. List of pilot sites/laboratories offering HPV primary screening in England prior to December 2018.

Location	Population converted at the time
Bristol	All
Cambridge	Part
Derby	Part
Kent and Medway	Part
Liverpool	All
London (Northwick Park)	Part
Manchester	Part
Norfolk Norwich	Part
Sheffield	Part

Note: Data was obtained from the National Cervical Screening Programme Manager at Public Health England

Demographic breakdown of step 2 sample with population comparison

In step 2 we set quotas for age, education and ethnicity based on the target population (Table S2). As we were unable to recruit enough participants with no qualifications, this category was combined with GCSE/O level grade for analyses. Quota categories set for ethnicity were white vs. non-white.

Table S2. Demographic breakdown of step 2 sample vs. population of English women aged 25-64

	Percent (n) in sample	Percent in population
	% (n)	%
Age[a]		
25-34	25.2 (152)	25.4
35-44	25.6 (154)	26.5
45-54	27.1 (163)	26.0
55-64	22.1 (133)	22.1
Education[a]		
No Qualifications	8.3 (50)	16.1
GCSE/O level grade or equivalent	40.5 (244)	32.3
A-levels or equivalent	15.0 (90)	11.8
Higher education or equivalent	36.2 (218)	33.6
Apprenticeship[b]	-	0.8
Other Qualifications[b]	-	5.5
Ethnicity[a]		
White	88.4 (532)	85.9
Mixed/Multiple Ethnic Groups	2.0 (12)	1.5
Asian/Asian British	6.6 (40)	8.0
Black/African/Caribbean/Black British	1.7 (10)	3.7
Other ethnic group	1.3 (8)	0.9
Social grade[a]		
AB (managerial/professional)	22.6 (136)	23.9
C1C2 (supervisory/skilled manual)	44.5 (268)	51.2
DE (semi-skilled/unskilled/unemployed)	32.9 (198)	24.9
Cervical screening experience[c]		
Yes	90.5 (545)	88.9[d]
No	8.8 (53)	10.9

Note: [a]Population data was obtained from the Office for National Statistics, Census 2011 data for England and Wales. Retrieved from: https://www.nomisweb.co.uk/census/2011/detailed_characteristics; [b] These categories were not included in our survey; [c]Population data represents test status in March 2018, and was obtained from NHS digital: https://digital.nhs.uk/data-and-information/publications/statistical/cervical-screening-programme/england---2017-18; [d] Includes 4.5% ceased for clinical reasons and 0.2% who never had an adequate sample. 0.2% of women had no cytology record.

Results for individual evaluation items in step 2

Table S3. Results for individual items assessing evaluations of the image depicting the speculum, the infographic depicting screening results, and overall evaluations of the leaflet

	Image speculum	Infographic results	Leaflet
	Mean (*SD*)	Mean (*SD*)	Mean (*SD*)
How much do you like or dislike this image/ the leaflet (1=do not like it at all–7=like it a lot)	4.4 (1.7)	5.8 (1.3)	5.6 (1.4)
How helpful or unhelpful do you think the image/leaflet is for deciding about whether to get a cervical screening test? (1=not helpful at all–7=extremely helpful)	5.1 (1.8)	6.0 (1.3)	6.1 (1.2)
How well do you understand the image/information in the leaflet? (1=do not understand at all–7=completely understand it)	6.2 (1.2)	6.3 (1.2)	5.9 (1.1)

Note: Items for the leaflet were worded in past tense. The full survey is available at https://osf.io/8wqzv/?view_only=6cd3279a3c0d466599a480c8d70de6e1

Results for composite measures (accuracy, confidence and evaluations) in step 2

Table S4. Mean accuracy scores, confidence ratings, and leaflet evaluations corresponding to the different levels of all predictors in regressions

	Accuracy of interpretations (0-23)	Self-reported confidence (50-100)	Leaflet evaluations (1-7)
	Mean (*SD*)	Mean (*SD*)	Mean (*SD*)
Age			
25-34	12.6 (3.1)	84.5 (9.8)	5.7 (1.2)
35-44	12.4 (3.0)	86.1 (10.2)	6.0 (1.0)
45-54	12.6 (3.0)	87.3 (10.0)	5.8 (1.0)
55-64	12.4 (3.1)	87.7 (9.5)	5.9 (1.0)
Education			
≤ GCSE/O level grade or equivalent	11.5 (2.8)	84.5 (10.6)	5.8 (1.1)
A-levels or equivalent	13.5 (2.9)	87.9 (9.7)	5.8 (1.1)
Higher education or equivalent	13.5 (3.0)	88.3 (8.6)	5.9 (1.1)
Ethnicity			
White	12.6 (3.1)	86.5 (9.8)	5.9 (1.0)
Non-white	11.9 (2.9)	85.4 (10.9)	5.8 (1.2)
Social grade			
AB (managerial/professional)	13.3 (3.2)	88.0 (9.5)	5.9 (0.9)
C1C2 (supervisory/skilled manual)	12.8 (2.9)	87.6 (8.8)	5.9 (1.1)
DE (semi-skilled/unskilled/unemployed)	11.5 (2.9)	83.6 (11.2)	5.7 (1.1)
Numeracy			
Score = 0	10.9 (2.5)	82.9 (12.8)	5.6 (1.4)
Score = 1	11.5 (2.7)	85.2 (11.1)	6.0 (1.0)
Score = 2	12.7 (2.7)	87.5 (8.4)	5.9 (1.0)
Score = 3	15.0 (3.0)	88.8 (6.9)	5.8 (1.1)
First language			
English	12.4 (3.1)	86.4 (9.9)	5.9 (1.0)
Other	13.0 (3.0)	85.8 (10.9)	5.6 (1.1)
Cervical screening experience			
Yes	12.5 (3.1)	86.8 (9.8)	5.9 (1.0)
No	12.6 (3.0)	82.0 (10.6)	5.4 (1.2)

Note: age and numeracy scores were entered as continuous variables in the regressions reported in the main text.

Responses for open-ended items assessing interpretations in step 2

Table S5a. Responses for item assessing estimates of the number of women expected to have an abnormal result

Imagine 1,000 women who have cervical screening. About how many of them will have an abnormal result? Correct answer: **60**		
Response	Frequency (n)	Percent (%)
**60**	**260**	**43.2**
6	184	30.6
1	47	7.8
94	13	2.2
50	8	1.3
40	7	1.2
100	7	1.2
2	6	1.0
20	6	1.0
600	6	1.0
Other	55	9.1
Missing	3	.5

Table S5b. Responses for item assessing estimates of the number of women expected to need treatment for abnormal cells

Imagine 1,000 women who have cervical screening. About how many of them will need treatment to remove abnormal cells? Correct answer: **20**		
Response	Frequency (n)	Percent (%)
500	114	18.9
**20**	**92**	**15.3**
40	63	10.5
4	51	8.5
6	46	7.6
60	33	5.5
2	32	5.3
50	31	5.1
1	28	4.7
10	19	3.2
30	19	3.2
100	17	2.8
3	7	1.2
5	7	1.2
200	7	1.2
Other	33	5.5
Missing	3	.5

Table S5c. Responses for item assessing estimates of the number of women expected to have possible cancer cells

Imagine 1,000 women who have cervical screening. About how many of them will have cells that could be cancer? Correct answer: **1**		
Response	Frequency (n)	Percent (%)
40	190	31.6
4	131	21.8
**1**	**62**	**10.3**
6	51	8.5
2	43	7.1
20	32	5.3
60	13	2.2
10	12	2.0
30	9	1.5
50	6	1.0
100	6	1.0
Other	44	7.3
Missing	3	.5

Table S5d. Responses for item assessing estimates of the number of women expected to get cervical cancer in a group of screened individuals

Among 1,000 women who do not have cervical screening, about 20 will get cervical cancer. Now imagine 1,000 women who do have cervical screening. How many do you think will get cervical cancer? Correct answer: **10**		
Response	Frequency (n)	Percent (%)
**10**	**209**	**34.7**
20	106	17.6
1	87	14.5
2	36	6.0
5	22	3.7
100	18	3.0
6	15	2.5
40	15	2.5
0	13	2.2
50	11	1.8
4	8	1.3
30	8	1.3
200	6	1.0
Other	46	7.6
Missing	2	.3

Figure S1 summarizes distributions of responses for open-ended items (Tables S5a-S5d), by categorizing incorrect responses as overestimations or underestimations.

Figure S1. Summary of responses for open-ended items. Missing responses are not shown.

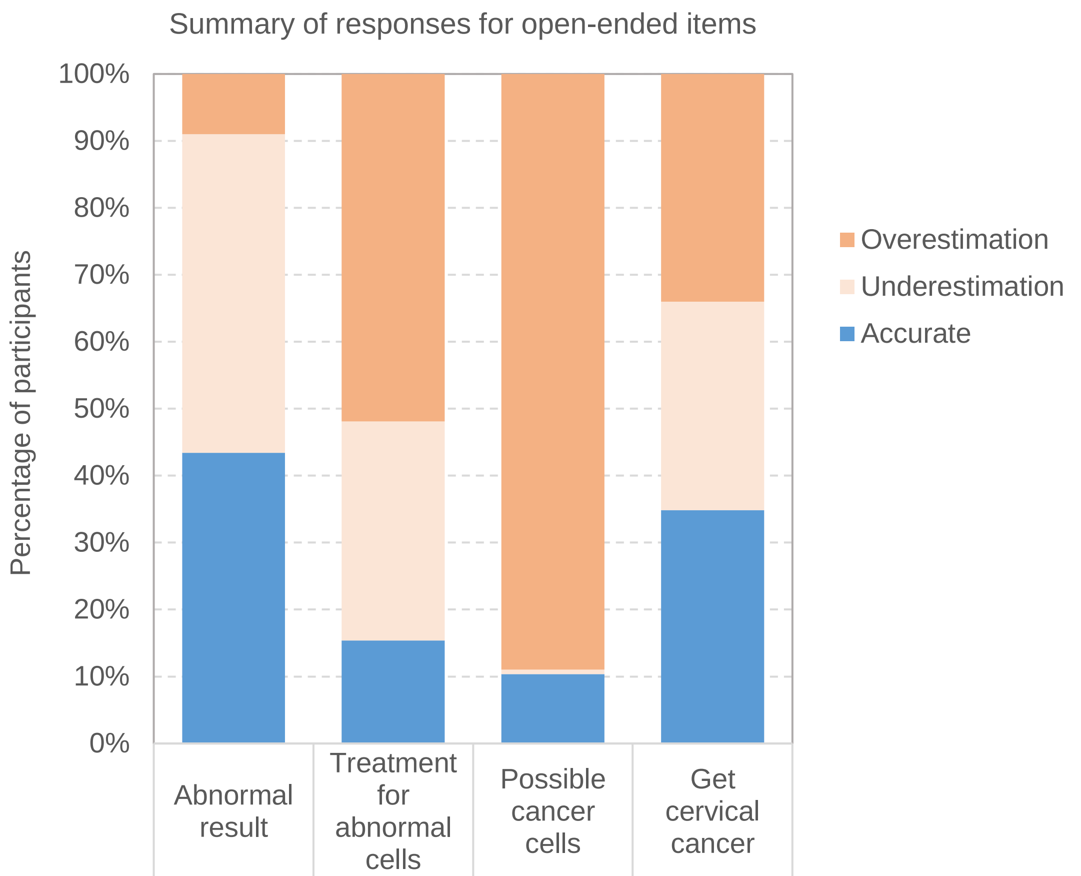

	Abnormal result	Treatment for abnormal cells	Possible cancer cells	Get cervical cancer
Overestimation	9.0	51.9	89.0	33.9
Underestimation	47.6	32.7	.7	31.1
Accurate	43.4	15.4	10.4	34.7

Note: The item “Get cervical cancer” (Table S5d) was designed to assess estimates of the effect of screening on the risk of getting cervical cancer. Underestimations of the number of women expected to get cervical cancer with screening reflect overestimations of the effectiveness of screening to reduce the risk. Conversely, overestimations of the number of women expected to get cervical cancer with screening reflect a perception of screening ineffectiveness or underestimations of screening effectiveness. Specifically, the category of overestimations includes 17.6% participants who inferred that the risk would be equal in groups of screened and unscreened individuals (i.e., answer=20) (ineffectiveness), 15.0% who inferred that the risk would be larger in the group of screened individuals (i.e., answer>20) (ineffectiveness), and 1.3% who inferred that the risk would be slightly smaller in the group of screened individuals (i.e., answer=11 to 19) (underestimation of effectiveness).
